# 3D Transparent Object Detection and Reconstruction Based on Passive Mode Single-Pixel Imaging

**DOI:** 10.3390/s20154211

**Published:** 2020-07-29

**Authors:** Anumol Mathai, Ningqun Guo, Dong Liu, Xin Wang

**Affiliations:** 1 School of Engineering, Monash University Malaysia, Jalan Lagoon Selatan, Bandar Sunway, Selangor 47500, Malaysia; anumol.mathai@monash.edu (A.M.); anthony.guo@monash.edu (N.G.); 2 State Key Laboratory of Modern Optical Instrumentation, College of Optical Science and Engineering, Zhejiang University, 38 Zheda Road, Hangzhou 310027, China; liudongopt@zju.edu.cn

**Keywords:** transparent object detection, single-pixel imaging, compressive sensing, disparity map acquisition

## Abstract

Transparent object detection and reconstruction are significant, due to their practical applications. The appearance and characteristics of light in these objects make reconstruction methods tailored for Lambertian surfaces fail disgracefully. In this paper, we introduce a fixed multi-viewpoint approach to ascertain the shape of transparent objects, thereby avoiding the rotation or movement of the object during imaging. In addition, a simple and cost-effective experimental setup is presented, which employs two single-pixel detectors and a digital micromirror device, for imaging transparent objects by projecting binary patterns. In the system setup, a dark framework is implemented around the object, to create shades at the boundaries of the object. By triangulating the light path from the object, the surface shape is recovered, neither considering the reflections nor the number of refractions. It can, therefore, handle transparent objects with a relatively complex shape with the unknown refractive index. The implementation of compressive sensing in this technique further simplifies the acquisition process, by reducing the number of measurements. The experimental results show that 2D images obtained from the single-pixel detectors are better in quality with a resolution of 32×32. Additionally, the obtained disparity and error map indicate the feasibility and accuracy of the proposed method. This work provides a new insight into 3D transparent object detection and reconstruction, based on single-pixel imaging at an affordable cost, with the implementation of a few numbers of detectors.

## 1. Introduction

Many practical activities in industry, such as automatic inspection, oceanology, fluid mechanism, and computer graphics, often require imaging of three-dimensional shapes of invisibles. Though various techniques have been developed for deducing 3D images of transparent objects, this class of objects poses difficulties due to many reasons. Firstly, these objects are colorless, and they gain a form from neighboring background objects. Secondly, the complexity of light interactions within the transparent objects makes its detection impossible. Finally, knowledge about the refractive index of the material is needed for reconstruction.

The existing techniques for transparent object inspection required application of known or unknown background (checkboard/striped) patterns in calibration with a camera, which is computationally a long process and costly. The first implementation of an unknown background pattern at the bottom of the water tank was performed to approximate the distorted water surface. Thereafter, structured light patterns were introduced to extract the surface properties of the glass objects [1]. Subsequently, direct rays were collected from the refractive objects by placing multiple cameras in several positions, with respect to the object to approximate the depth [2]. Later, the shape of transparent objects was reconstructed from its known motion [3]. Dusting and submerging transparent objects in fluorescent liquids deteriorated the structure of the object, therefore, it has limited its implementation in real-time applications [4,5]. Presently, transparent object recovery is achieved by combining polarization analysis and light-path triangulation [6]. Almost all the techniques for depth acquisition rely on some external factors, such as system calibration, background patterns, less intensity environment, and object motion, which will introduce a lot of errors to the inspection system. Hence, the accuracy is relatively low.

In addition, a range of sensors, such as color cameras, light detection and ranging (LIDAR) systems, time of flight (TOF) cameras, IR cameras and Kinect sensors, have been developed to image transparent objects, yet a general solution could not find for the detection of transparent objects [7,8,9]. In color cameras, object recognition can be performed when the background color of the object is exactly the same as the color of the object. When LIDAR/TOF sensors are used to image a transparent object, the reflected light from the object has recorded an approximate shape. The projected light from these sensors is reflected multiple times before it hits the transparent object, which does not cause object information in the camera. Hence, the shape cannot be recognized, and the edges of the object have been missed, which made object reconstruction impossible. Kinect sensor is an alternative method utilized for transparent object inspection, in which a sensor is moved in the scene to acquire multiple views of the object [7]. Additionally, the work is limited to non-planar transparent objects with a smooth surface. With an IR camera, Maldague et al., proposed a technique called “shape from heating”, in which the transparent object surface was heated with a thermal camera source, and the time sequence of thermograms (thermal images) was recorded to estimate the shape [10]. Later, Eren et al. developed “scanning from heating” to detect 3D transparent objects on the basis of “shape from heating” technique, used by Maldague et al. [11]. In this work, the object was heated with a laser source, to a temperature at which the object turns opaque and then, irradiations were recorded by a thermal camera for shape estimation. The main limitations of these studies were the non-uniform surface heating of the object and the use of an infrared laser source, which restricted the performance of both the studies. The identified limitations in the sensors can be solved with an alternative sensor called a “single-pixel detector”.

Single-pixel imaging (SPI) is an advanced imaging approach that is applicable for acquiring spatial information in low light, high absorption, and backscattering conditions [12]. SPI has been widely used in myriad applications, such as infrared imaging [13], gas imaging [14], photoacoustic imaging [15], three-dimensional imaging [16,17,18], terahertz imaging [19,20], X-ray diffraction tomography [21], remote sensing [22], encrypted imaging [23], lensless imaging [24], shadowless imaging [25], hyperspectral imaging [26], microscopy [27], and scattering imaging [28]. In this imaging modality, a single-pixel detector that has no spatial resolution can detect the object by means of a modulated structured light [29]. Though this imaging technique is affected by noises, the ability to work in challenging environments with high resolution and precision enables single-pixel detection more popular than any other conventional imaging systems [30]. Moreover, its sensitivity to a wide operating spectrum extends its operation range beyond the visible spectrum [31]. All these characteristics of single-pixel imaging have been utilized to recover the images from challenging environments. The smart control of light propagation with prior knowledge of the modulated patterns in this technique moderates the depletion of the ballistic photons. Tajahuerce et al. proposed an optical system using a single-pixel camera, which can successfully reconstruct 2D objects, even under multiple scattering conditions in turbid media [28]. In addition, this work compared the quality of 2D image reconstruction with the result of CCD camera. In the presence of a scattering medium in front of the object, a CCD camera can only capture the speckle pattern (no information regarding the object). On the contrary, a single-pixel detector can record an excellent result. Based on the modulation involved, this technique is classified as active and passive single pixel imaging. Both methods are implemented in many imaging modalities for the acquisition of 2D and 3D objects.

Magalhães et al. presented an active illumination single-pixel camera, in which a photodetector approximated a replica of the object by averaging the inner product between the pattern and the object sample [32]. The problem of spatial and temporal aberrations that occurs in imaging transparent object was resolved in [12]. Later, Bertolotti et al. [33] proposed a non-invasive imaging technique that uses an iterative algorithm to retrieve the image of a fluorescent object hidden in the opaque medium. There are many published works on non-line of sight imaging, in which the object is sandwiched between two layers of chicken pieces with a thickness of 2.84 mm and 2.92 mm [34,35]. The imaging has performed using a single-pixel detector and estimated the image of the sandwiched object. Winters et al. recommended a method to improve the speed of reconstruction in scattering media with the help of help of x and y modulators. These modulators can operate at extremely high speed control the illumination pattern before sampling the object [36]. All the above-mentioned works give an insight into the reconstruction of 2D objects.

Three-dimensional image reconstruction approaches, such as time-of-flight (TOF), binocular vision, photometric stereo, and shape-from-X techniques can estimate the depth information of opaque objects. Sun et al. [37] proposed a model based on the “shape from shading” method, where multiple cameras were placed at different positions of the reflective object. Two-dimensional images from each camera hold the shadows of the object from which surface gradients are derived and 3D images are reconstructed via photometric stereo. Wen-kai et al. [38] developed a 3D reconstruction system using a binocular stereo vision algorithm and a single-pixel detector by placing the object on a rotating platform. The limitations of using spatially separated multiple detectors and moving the object were eliminated in [17]. The TOF technique is utilized to obtain the depth information and its accuracy depends on a high-speed photodiode and precision in measurements. Similarly, other demonstrations for scanning the scene and obtaining the depth and reflectivity information via TOF have also been discussed [39,40,41,42]. Zhang et al. proposed a method to capture the images of opaque objects. In this study, four photodetectors were implemented at different locations of the object to capture the reflected light. The variations in shading information in the images were studied, and a photometric stereo algorithm was utilized for 3D image reconstruction [43]. Salvador-Balaguer et al. [44] implemented a basic active single-pixel imaging system to image opaque objects. They also used reflected light from the object and processed it, based on an adaptive compressive algorithm for image reconstruction. All these methods are suitable for recovering 3D images of objects for reflective surfaces. The perfect assembly and tuning of all instruments with high precision are needed to achieve the target reconstruction. If the relevant parameters of each instrument in the system are properly raised, and the high-precision assembly is matched, all these techniques can assure the overall reconstruction quality of the system.

In this paper, we present a fixed multi-viewpoint 3D transparent object inspection system, based on passive mode single-pixel imaging. Two single-pixel detectors are applied in the setup to eliminate the need to move the object while imaging. The results show that it is possible to obtain the disparity map of the object by using high-speed detectors to record the sampled refracted light, along with our image reconstruction algorithm. The rest of the paper is organized as follows. Section 2 describes our experimental setup for 3D transparent object detection. Section 3 examines how 3D depth information is extracted from 2D images. This is followed by the conclusion, which is expounded in Section 4.

## 2. Experimental Setup

**Figure 1:**
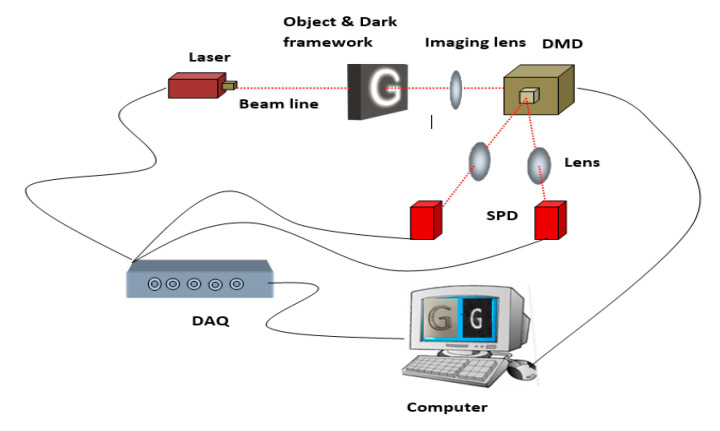
Schematic of 3D transparent object detection and disparity map acquisition system. The red dashed line indicates the laser light beam from the source to detectors.

The schematic diagram of the proposed 3D transparent object detection system is shown in the Figure 1. The system consists of a red laser to illuminate the transparent object, a dark framework to cause streak effects at the object boundary, an imaging lens, a digital micromirror device (DMD) to modulate the laser light with a computer-generated measurement matrix, collecting lenses, two single-pixel detectors to collect the transmitted light from the object, a data acquisition (DAQ) system, and a computer, to perform 2D and 3D image reconstruction.

**Figure 2:**
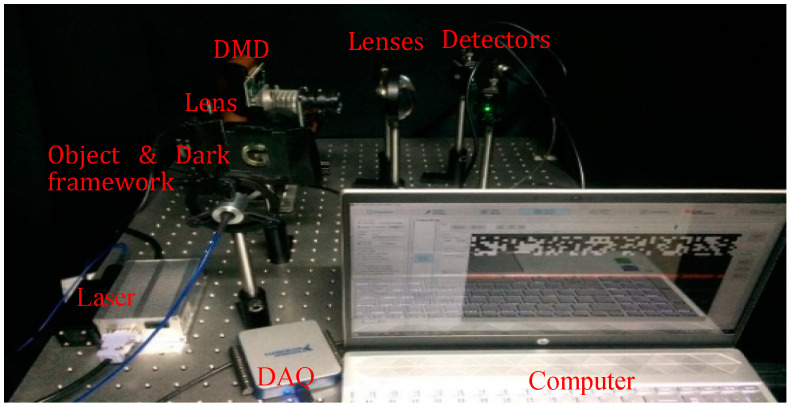
Experimental setup implemented in our lab environment.

The experimental set up is shown in the Figure 2. The fiber output red laser (650 nm, 1 W) is to illuminate the target object. The refracted light from the target is collected by an imaging lens (LA1740, f-85 mm) and directed to DMD micromirror (DMD6500 &9000) active area. To provide spatial information to the captured image, the pre-programmed patterns stored in the DMD is combined with the transmitted light. Then, the modulated light from the DMD is projected to the environment, where two focusing lenses collect the light and the light is focused to the active area of the spatially unresolved single-pixel detectors. The pre-programmed patterns in the experiment provide spatial information to the transmitted light. The experimental setup is employed for passive modulation mode where two PDA36A2- photodetectors are used as single-pixel detectors to record the total light intensity from the object. DAQ(USB6001) digitizes the recorded light from left and right single-pixel detectors, and sends it to a computer to conduct 2D image reconstruction. The 2D image quality depends on patterns used and any distortions in it deteriorate the image quality. While choosing the passive mode, the intensity transformed object information is modulated with patterns in the DMD, which will reduce the distance at which modulated light beam travels, thereby reducing the distortion through ambient light. Any deviations in pattern structure can also be maintained in the passive method.

For a single-pixel imaging system, the orientation of the optical components and specifications of the lenses often play a crucial role for ensuring high quality images. Additionally, the proper selection of the lens and its focal length is essential to concentrate the transmitted light beam from the object to the very small active area of the single-pixel detector. Furthermore, combining lenses such as planar and aspheric ensures sharp focus with fewer aberrations, resulting in better quality 2D images. As disparity accuracy is closely related to the quality of the 2D reconstruction result, the lens must be chosen cautiously. The proposed experimental setup is implemented in the lab environment with a black framework around the sides of the object to provide a streak effect on the edges of the object. The disparity calculation will depend on the quality of 2D images and edge sharpness. In our work, transmitted light (majority of the light) is collected for image reconstruction, which will provide good quality 2D images compared to conventional methods. Moreover, the features of a single-pixel detector, such as increased detection efficiency, lower noise, and higher time resolution etc., provide additional advantages. Additionally, the apparent directional illumination from DMD and shadow effect at the edges of the object make the system superior in producing good quality 2D images. After obtaining left and right single-pixel detector images, the 3D reconstruction algorithm first looks for the preserved edges, and then finds out the disparity between the pixels for depth calculation.

Additionally, calibration of the left and right single-pixel detector images is required to maximize the accuracy in disparity map calculation, because the 2D images are taken from different angles of the object. In the calibration process, multiple images of the object are captured from different perspectives, and self-calibration is performed to obtain the intrinsic and extrinsic parameters for depth calculation [45,46]. To ensure accurate measurement, the trigger signal is set to initiate the DMD to modulate the incoming light with preprogrammed patterns. The exposure time and dark time of the DMD are decided by the number of samples to be recorded for a period. So, the calibration process reduces the probability of error and consistently increases the measurement process systematically.

For the experimental setup, two single-pixel detectors are synchronized, such that both the detectors can capture images at the same time when DMD project patterns. The number of samples to be recorded is set as 100 in a second for each displayed pattern. DAQ takes an average of 100 samples to obtain a single measurement that corresponds to each pattern and sends it to a high-performance computer for further processing. This operation will continue until the DMD stops pattern projection. In addition, the detectors are placed with a distance of 7 cm between them, to obtain the full view of the object. The distance from the camera to the object is set as 65 cm, and the focal length of the single-pixel detector is set as 8.5 cm, which is based on the focal length of the lenses used for focusing the light on the single-pixel detector.

## 3. 3D Transparent Object Reconstruction

### 3.1. 2D Image Reconstruction

**Figure 3:**
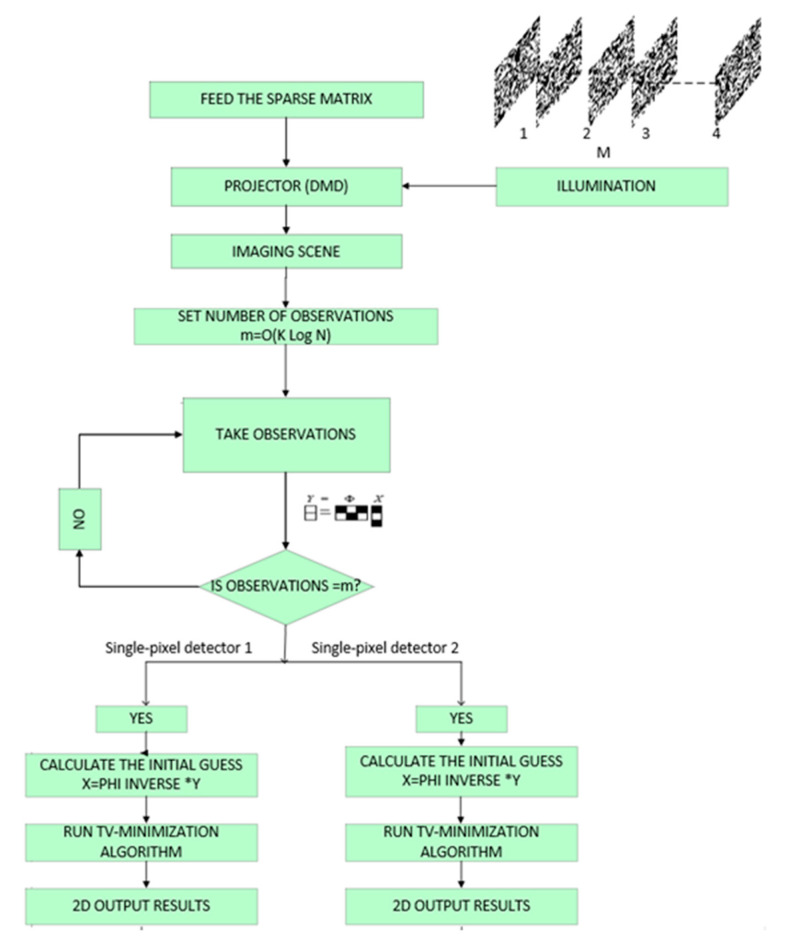
Flowchart of transparent object detection process.

**Figure 4:**
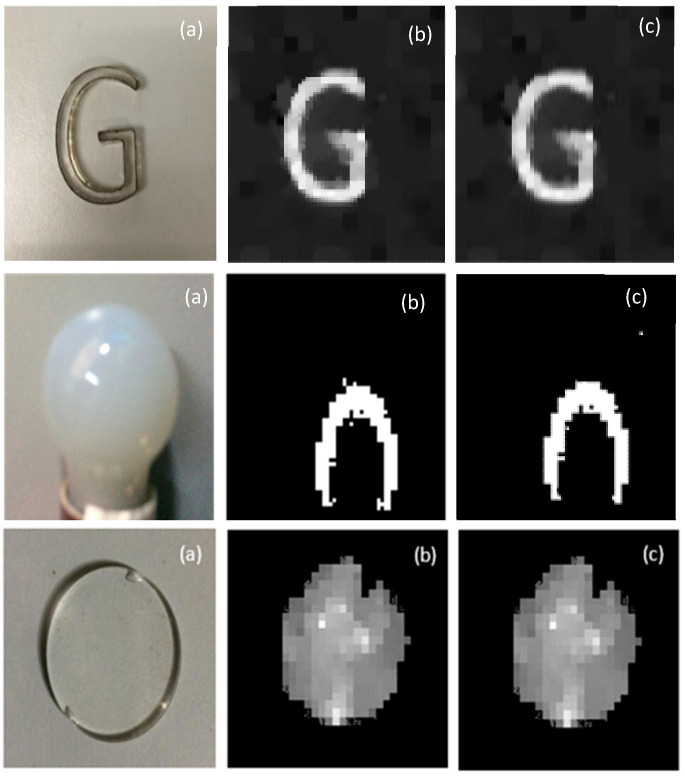
Two-dimensional image reconstruction results for the passive single-pixel imaging method. (**a**) The original object for reconstruction: “G” has a thickness of 10 mm, “bulb” has a size of 20×26 mm and “Transparent-circle” has a thickness of 5 mm. (**b**) The reconstructed 2D image from left and (**c**) right detectors.

The advent of DMD and single-pixel imaging enables fast image reconstruction with a few measurements. The transparent object detection and image acquisition process are shown in the Figure 3. The object to be imaged is fixed at a position and it is scanned and sampled with a sequence of the sparse matrix (up to M numbers). The resolution of the reconstructed image is decided based on the resolution and number of the projected sparse matrix. For the following step, the measurements required for reconstruction is fixed to m (m=O(KlogN)), where the total number of pixels in the object is *N*, due to the adoption of compressive sensing (CS) in acquiring the samples. The detectors in the imaging system collect object samples, until DMD stops matrix pattern projection. The number of patterns projected depends on the sparsity of the measurement matrix. At last, the total variation (TV) minimization algorithm estimates the original signal X from the measurement vector Y with the prior knowledge of the sparse matrix. The result obtained from the system is displayed in Figure 4.

Recovering an image, $X_{N\times N}$ from a set of measurements vector, $Y_{N\times N}$ is straightforward with matrix inversion techniques. With this technique, single-pixel imaging had limitations, such as the requirement of $N^{2}$ pixels for reconstruction, long data acquisition time, and large data storage. These problems can be addressed by combining single-pixel imaging and compressed sensing. It enables the single-pixel detector to reduce the number of measurements required for reconstruction to $Y_{M\times 1}$, thereby reducing data storage and data transfer requirements. This method also solves a linear inverse problem in the case where X has a sparse representation.

The SPI technique gathers light, which interacts with the object with the aid of a spatially un-resolved single-pixel detector. The encoding of spatial information in the collected light is done by the pre-programmed spatially resolved patterns. The single-pixel detector sequentially measures the inner products between the N×N pixelated scene and a set of M×N binary patterns. The principle behind the CS imaging is summarized in equation [47]:

(1) Y=ΦX

where Y is an M×1 column vector, Φ is the measurement matrix contains, M is the row vector, N is the column vector, and X is the representation of original image, having N×1 pixels. When the number of measurements M in Y is less than the total number of pixels (N) in X, the Equation (1) will become an ill-conditioned problem with infinite solutions. To solve such problems, the original image should obey the property called sparsity, in which only the most significant co-efficients (K-sparse) in the image are considered for processing, and all the less significant co-efficients are discarded. In CS, the K-sparse information is acquired and stored in the column vector Y. If an image can be represented in some basis, then it can be recovered via $l_{1}$ minimization, with the knowledge of Y and Φ [47].

Consider a K-sparse signal X and it is sparse in orthogonal basis, $\Psi =\left[\Psi_{1},\Psi ,{}_{2}\ldots \Psi_{N}\right]$, then

(2) X=ΨS

where S is K-sparse, in which K coefficients are non-zero. According to CS theory, the signal X an be recovered with m(m=O(KlogN)) incoherent linear measurements when the original signal contains such K-sparse co-efficients. Then, Equation (1) becomes:

(3) Y=ΦX=ΦΨS

where Φ s a pre-programmed pattern of the size M×N, which is uncorrelated with the sparsity basis Ψ, and Y is the M×1 measurement vector [48]. From the measurement vector Y, image recovery is achieved by the TV-based minimization model. The directional change (gradient) in the object image X can be determined at a pixel location $x_{ij}$ [49]:

(4) $$\begin{array}{l} G_{ij}=\left(\begin{array}{c} G_{h;ij}(X)\\ G_{v;ij}(X) \end{array}\right)\\ G_{h;ij}(X)=x_{i+1,j}-x_{i,j}\\ G_{v;ij}(X)=x_{i,j+1}-x_{i,j} \end{array}$$

The TV minimization algorithm calculates the total variation and removes the undesirable information by preserving the edges at each pixel location of the image X:

(5) $$TV(X)=\sum_{ij}\sqrt{G_{h;ij}{\left(X\right)}^{2}+G_{v;ij}{\left(X\right)}^{2}}$$

TV minimization has been adopted for most image processing fields due to its ability to keep visual quality than $l_{1}$ optimization [49]. To acquire the 2D image of the size 32×32, the conventional imaging system would take 1024 measurements. In SPI, 200 measurements, around 20% of the total number of pixels, are used for good quality image reconstruction. Three objects are tested, and the resultant images reconstructed from the left and right single-pixel detectors are shown in Figure 4.

Our experimental setup contributes to the formation of good quality 2D images for transparent objects along with CS algorithm. The 2D image reconstruction quality obtained from the SPI system is better than the conventional imaging systems, such as LIDAR or TOF cameras [7,8,9], owing to single-pixel sensors detection efficiency, lower noise, and higher time resolution. The apparent directional illumination from DMD and shadow effect at the edges of the object also make the system superior to traditional imaging methods in obtaining good quality image reconstruction.

### 3.2. Disparity Map Using Normalized Cross-Correlation (NCC)

The object in the experimental setup is observed by two single-pixel detectors. This is equivalent to getting images of the object from two angles without changing the position of it. Binocular stereovision determines the position of a point in space by finding the intersection of two lines passing through the center of projection and the projection of point in the image. The images from the two viewpoints are dissimilar in intensity distribution and the depth information of the images is lost. However, depth can be inferred through the binocular vision algorithm, which works very similarly to human eyes. Stereovision algorithms are classified as features based and window/area-based techniques. Feature based algorithms are complex in finding the matching features for all the edges or corners from two single-pixel images to build a disparity map. Thus, the area-based method is considered for depth evaluation, in which the algorithm matches blocks of pixels to find correspondences in the images. In this study, the NCC method is used to determine the correspondence between two windows around a pixel of interest. NCC is defined as:

(6) $$NCC\left(i,j,d\right)=\frac{\sum_{\left(i,j\right)\in w}X_{l}\left(i,j\right).X_{r}\left(i^{’}-d,j’\right)}{\sqrt{\sum_{\left(i,j\right)\in w}X_{l}{}^{2}\left(i,j\right).\sum_{\left(i,j\right)\in w}X_{r}{}^{2}\left(i^{’}-d,j’\right)}}$$

where w is the window size, $X_{l}$ is the left detector image, $X_{r}$ is the right detector image, and d is the disparity. i,j and $i^{’},j^{’}$ are the blocks of pixels to be matched in the left and right detector images, respectively. The window size can affect the quality of the disparity map, in this work we have chosen window size as 6×6 pixels.

**Figure 5:**
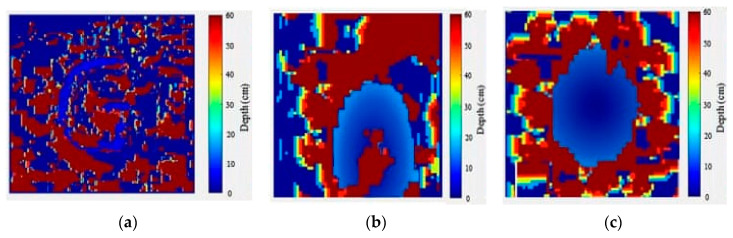
Disparity map acquisition of 3D transparent objects based on single-pixel imaging. (**a**) The depth map of the object “G” is displayed with a disparity range. (**b**) The depth map for the complex object is illustrated with a disparity range. (**c**) The depth map for the object “Transparent-circle”.

Three-dimensional image reconstruction quality depends on the quality of 2D images and its edge sharpness. The complete details about the edge features aid in estimating the boundaries of the object from the background. Two-dimensional images obtained from SPI are noisy and edges are not uniform. Hence, background noise has been removed first, and then the canny operator algorithm has been applied for edge detection. After that, the image is processed using morphological operators to make the edges smooth and perfect. The major difficulty for transparent object detection is the featureless surface to compute the disparity. This issue is resolved to some extent in this work, owing to the tracing of edges and the significant role of the NCC algorithm in depth computation. As the NCC algorithm is less sensitive to the changes in the intensity value in each pixel, the depth computation with the algorithm become more precise. Depth information from a pair of images can be calculated by first computing the distance between the block of pixels at a location in the left image and its corresponding location in the right image. The search for the best match is performed over a window. This will produce a disparity map, as shown in Figure 5. Before the disparity calculation, the left and right images are converted to grayscale images. Hence, the NCC algorithm determines the intensity range in the images, normally between 0 and 255, and divides the range into multiple offsets (from 0 to 30 offsets), having a range of pixel intensities within each offset. At the same time, the offset adjust is also calculated using the given formula:

(7) $$Offset\;adjust=\frac{255}{30}$$

where *offset adjust* is used in the final step of the algorithm to calculate the pixel range. For NCC calculation, the left image is fixed, and the right image is moved across the window and intensities of both left and right images get multiplied and further divided by their own intensity square or standard deviation of the intensities across the window. Then, the disparity is calculated using the following equations:

(8) $$\begin{array}{l} M=\sum_{i,j}\sum_{i,j}X_{l}\left(i,j\right).*X_{r}\left(i^{’}-d,j^{’}\right)\\ X_{r}{}^{2}=\sum_{i,j}\sum_{i,j}X_{r}\left(i^{’}-d,j’\right).*X_{r}\left(i^{’}-d,j’\right)\\ X_{l}^{2}=\sum_{i,j}\sum_{i,j}X_{l}(i,j).*X_{l}(i,j) \end{array}$$

(9) $$NCC=\frac{M}{\sqrt{\left(X_{r}^{2}*X_{l}^{2}\right)}}$$

where M represents the dot product between the left and right images. The similarity of the pixels from both left and right images is aggregated over the window size as shown in Equation (6) ariations in window size will affect the quality of the reconstructed images. Increased window size makes the disparity map smoother, however, there will be inaccuracies in object detailing at the boundaries. Hence, smaller window size is chosen to provide maximum depth details with more noise. The obtained disparity from the above equations is multiplied with the offset value to get the pixel range which is given by the equation:

(10) Disparity map=disparity∗offset adjust

Depth “z” of the object from the photodetector is calculated using the following formula:

(11) $$z=\frac{b\times f}{d}$$

where b represents the baseline distance i.e., the distance from the optical center of one detector to other, f symbolizes the focal length of the photodetector, and d indicates the disparity between the pixels in one image to another image.

The disparity map of the object is plotted in Figure 5 for various objects. The depth bar, which is in cm, indicates the distance at which object is placed from the camera. From the depth bar, the pixel value within the minimum offset range would indicate the farthest away information (background) in the plot, or else the pixel value within the maximum offset range would indicate the nearest information from the detector in the plot. The widely accepted bad matched pixel (BMP) measure is used to quantitatively evaluate the disparity maps for error estimation, and it is calculated using following formula:

(12) $$\begin{array}{ll} BMP=\frac{1}{N}\sum_{\left(x,y\right)}\varepsilon (x,y);\;\varepsilon (x,y)=1\;\mathrm{if}\; & \left|D_{\mathrm{true}}\left(x,y\right){-D}_{\mathrm{reconstructed}}\left(x,y\right)\right|>\delta \\ \;0\;\mathrm{if}\; & \left|D_{\mathrm{true}}\left(x,y\right){-D}_{\mathrm{reconstructed}}\left(x,y\right)\right|<\delta \end{array}$$

where $D_{\mathrm{true}}$ represents the ground truth data, and $D_{\mathrm{reconstructed}}$ represents the disparity map data. The error tolerance value δ is commonly taken as 1. The BMP value computed for the three disparity maps in Figure 5a–c is given by 0.221, 0.218, and 0.202, respectively. The values obtained are the measure of the quantity of errors occurring in disparity maps.

**Figure 6:**
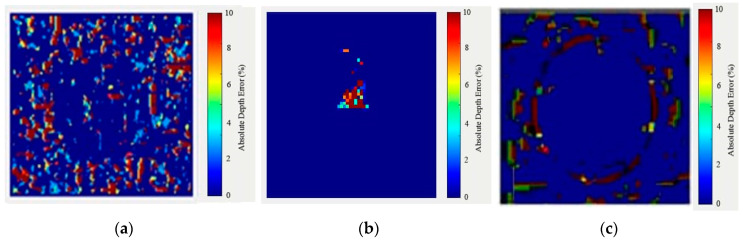
The error map computation result for the object (**a**) “G” (**b**) “bulb” and (**c**) “Transparent-circle”.

To verify the effectiveness of our method, the reconstructed images are aligned with the original ground truth images to compute the error map shown in Figure 6. The percentage error (%) is calculated from the difference between the ground truth image and reconstructed image. The equation for calculating the percentage depth error is given by the formula:

(13) $$\;Absolute\;depth\;error(\%)=\frac{\left|ground\;truth\;image-\;reconstructed\;image\right|}{ground\;truth\;image}\times 100\;\%\;$$

The Otsu method is implemented by setting the threshold value as one while comparing the ground truth image with the reconstructed image.

The results show that the proposed transparent object inspection system works very well in capturing images and finding the disparity. When comparing our work with existing techniques, the proposed system is superior in reconstructing the shapes under visible light with cost effective single-pixel detectors. In addition, the movement of object or the camera in the scene for acquiring multiple views of the object is not needed in the proposed setup. Moreover, images are reconstructed with a smaller number of measurements, due to the application of CS, thereby reducing the storage requirements and time-consuming computations. Some parts of the objects are not detected in the reconstructed results, because of fewer transmissions from the object. Additionally, the quality of the 3D reconstruction results is not as good as expected, due to some missing parts in reconstructed 2D images. Post-processing of the 2D images is necessary before feeding the 2D images for the disparity calculation program. Moreover, an increase in window size to obtain the finer details of the reconstruction adds more noise into the image which causes the 3D image to become blurrier and noisier.

## 4. Conclusions

In conclusion, we have experimentally demonstrated a 3D transparent object inspection system with two single-pixel detectors by collecting the transmission from the object. The employment of two single-pixel detectors overcomes the limitation of object movement during imaging. Two-dimensional images are reconstructed using convex optimization algorithms based on CS. The employed NCC algorithm successfully deduced the depth map from the 2D image. The resultant 3D image using the proposed passive single-pixel imaging setup and NCC algorithm ensures better quality, compared to conventional imaging methods. The system developed for transparent object inspection can detect objects with flat and homogeneous surfaces with limited thickness. More experiments will be conducted for complex objects, and the 3D image reconstruction algorithm will be further improved in the future.
